# Positive and Negative Regulation of Cellular Immune Responses in Physiologic Conditions and Diseases

**DOI:** 10.1155/2012/485781

**Published:** 2012-03-26

**Authors:** S. Viganò, M. Perreau, G. Pantaleo, A. Harari

**Affiliations:** ^1^Division of Immunology and Allergy, Department of Medicine, Lausanne University Hospital, 1011 Lausanne, Switzerland; ^2^Swiss Vaccine Research Institute, 1011 Lausanne, Switzerland

## Abstract

The immune system has evolved to allow robust responses against pathogens while avoiding autoimmunity. This is notably enabled by stimulatory and inhibitory signals which contribute to the regulation of immune responses. In the presence of a pathogen, a specific and effective immune response must be induced and this leads to antigen-specific T-cell proliferation, cytokines production, and induction of T-cell differentiation toward an effector phenotype. After clearance or control of the pathogen, the effector immune response must be terminated in order to avoid tissue damage and chronic inflammation and this process involves coinhibitory molecules. When the immune system fails to eliminate or control the pathogen, continuous stimulation of T cells prevents the full contraction and leads to the functional exhaustion of effector T cells. Several evidences both *in vitro* and *in vivo* suggest that this anergic state can be reverted by blocking the interactions between coinhibitory molecules and their ligands. The potential to revert exhausted or inactivated T-cell responses following selective blocking of their function made these markers interesting targets for therapeutic interventions in patients with persistent viral infections or cancer.

## 1. Introduction

The immune system has evolved to allow robust responses against pathogens while avoiding autoimmunity. This is notably enabled by stimulatory and inhibitory signals which contribute to the regulation of immune responses. Positive costimulation is critical for the development of T-cell immune responses against foreign pathogens, while negative regulation is critical for the termination of immune responses, for peripheral tolerance, and to avoid inflammation-induced tissue damage [1–3].

When self/nonself antigens discrimination fails or when invading pathogens are not controlled, the immune system starts destroying cells and tissues of the body and consequently causes autoimmune diseases and chronic syndromes. In this regard, costimulatory and coinhibitory molecules are involved in regulating the initiation and termination of T-cell responses as well as spontaneous autoimmunity [3–5].

T-cell activation is determined by the presence of three distinct signals: (1) TCR-MHC class I and II interaction, (2) costimulatory molecules interaction, and (3) cytokines signaling. In the past, the dogma, based on initial observations, was that the integration of the distinct signals triggered T-cell activation, whereas the lack of complete positive signals led to tolerance or anergy [6–8]. More recently, the complexity of the model increased following the discovery of coinhibitory molecules triggering inhibitory signals. The functional outcome of costimulatory and coinhibitory molecules signaling is either enhancement or inhibition of TCR-mediated immune responses [9].

**Figure 1:**
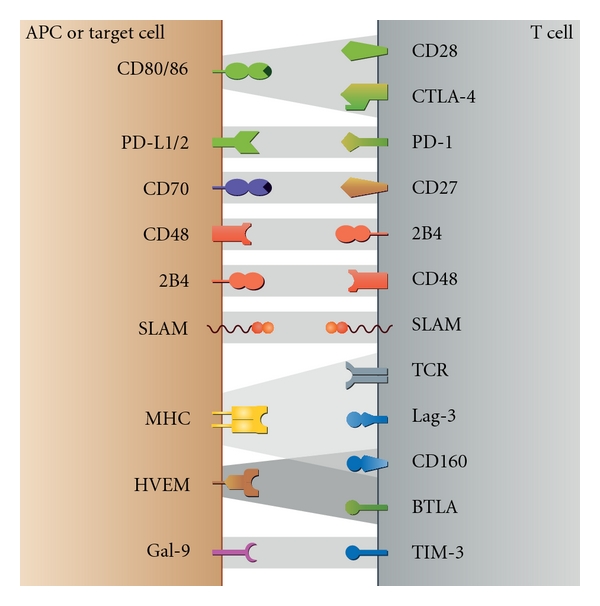
Regulatory molecules and their ligands. Schematic overview of the different costimulatory and coinhibitory molecules expressed by T cells (right panel) and association with their respective ligands expressed by antigen-presenting cells (APCs) or target cells (left panel). Coinhibitory molecules are color coded according to their relevant families. The four families of regulatory molecules include (1) B7-CD28 family including CD28, cytotoxic T-lymphocyte antigen-4 (CTLA-4; CD152), programmed death-1 (PD-1; CD279), inducible costimulatory molecule (ICOS; CD278), and B- and T-lymphocyte attenuator (BTLA; CD272); (2) CD2/signaling lymphocyte activation molecule (SLAM) family including SLAM (CD150), 2B4 (CD244), and CD48; (3) Ig family including T-cell immunoglobulin mucin-3 (TIM-3), CD160, and Lymphocyte-activation gene 3 (Lag-3); and (4) TNF-receptor superfamily including CD27.

Over the past decade, four different families of costimulatory and coinhibitory molecules able to modulate TCR signaling have been identified: (1) B7-CD28 family including CD28, cytotoxic T-lymphocyte antigen-4 (CTLA-4; CD152), programmed death-1 (PD-1; CD279), inducible costimulatory molecule (ICOS; CD278), and B- and T-lymphocyte attenuator (BTLA; CD272) [1]; (2) CD2/signaling lymphocyte activation molecule (SLAM) family including SLAM (CD150), 2B4 (CD244), and CD48 [10, 11]; (3) Ig family including T-cell immunoglobulin mucin-3 (TIM-3) [12, 13], CD160 [14, 15], and lymphocyte-activation gene 3 (Lag-3) [16]; and (4) TNF-receptor superfamily including CD27 [17] (Figure 1).

In the presence of a pathogen, a specific and effective immune response must be induced and naïve T cells undergo activation upon encounter with their specific antigens [18, 19]. This leads to antigen-specific T-cell proliferation [20, 21], cytokines production, and induction of T-cell differentiation toward an effector phenotype [22] combined to survival signals [23, 24]. After clearance or control of the pathogen, the immune response must be terminated in order to avoid tissue damage and chronic inflammation [24, 25]. Two main mechanisms are involved in the contraction of the effector phase of immune responses, that is, either the inhibition of T-cell expansion [26] or the elimination of activated cells by apoptosis [27]. The latter is referred to as activation-induced cell death (AICD) [27, 28]. Direct inhibition of T-cell proliferation is induced via signals through coinhibitory molecules such as CTLA-4 or PD-1, while 2B4 and SLAM are considered to be critical in the regulation of AICD [29].

The role of coinhibitory molecules in regulating the immune system is also evidenced by severe autoimmune and lymphoproliferative diseases resulting from the lack or aberrant expression of these molecules [30].

## 2. Expression of Coinhibitory Molecules on Effector T Cells

T cells play an important role in the defense against infectious agents and tumors. Upon recognition of their cognate antigen, naïve T cells get activated and differentiate into effector cells [31]. This activation results in both phenotypic and functional changes that will determine the fate of effector T cells and the efficacy of the immune response [22]. Several studies have aimed to better define the profile of effector cells associated with the efficient control of infectious agents or tumors [32, 33]. While most studies focused on the differentiation state or functional profile of effector cells [34, 35], a lot of attention has been paid recently to the role of coinhibitory molecules [36].

SLAM family members, for instance, are immunomodulatory receptors associated with different functions including costimulation, cytokines production, and cytotoxic activity of immune cells (e.g., T cells or NK cells). Most members of this family serve as their own ligand on target cells or interact with molecules from the same family (e.g., CD48 and 2B4) [37, 38] and signal through a common messenger, that is, the SLAM-associated protein (SAP). SAP consists almost entirely of a single SH2 protein domain interacting with the cytoplasmic tail of SLAM and related receptors. One member of this family is SLAM and its signaling is involved in the induction and regulation of CD8 T-cell effector functions. In particular, SLAM is involved in the induction of IFN-*γ* production, cytotoxic activity, proliferation capacity, and activation-induced cell death (AICD) of activated CD4 and CD8 T cells [39–46]. In normal CD4 and CD8 T cells, SLAM enhanced TCR-mediated cytotoxicity and IFN-*γ* production [47], in contrast, T cells from SLAM-deficient mice showed increased IFN-*γ* production upon stimulation, thus supporting a negative role for SLAM in the regulation of IFN-*γ* production by effector T cells [10, 48, 49]. 2B4 (CD244), which is another important member of SLAM family, is a cell-surface glycoprotein structurally related to CD2-like molecules such as CD2, CD48, CD58, CD84, and Ly-9 [50] and seems to be particularly important for the cytotoxic effector function of CD8 T cells and NK cells [51–55].

Regarding the Ig family, CD160 which binds to classical and nonclassical HLA I molecules (i.e., HLA class Ia/b, HLA-C on NK cells) and Herpesvirus Entry Mediator (HVEM) [56], was identified not only on most NK cells and *γδ* T cells but also on a subset of CD8 *αβ* T cells [57, 58]. In NK cells, CD160 engagement induces cytotoxicity [59, 60] and has also been characterized as a marker of cytotoxic effector CD8 T cells [15, 61, 62]. However, the expression of CD160 on effector CD8 T cells is more controversial since no association between CD160 expression and perforin content was observed in CMV-specific CD8 T-cells [63] whereas, in HIV-1-infected patients, a CD160^+^CD8^high^ effector T cell subset containing high amount of granzyme B (GrmB) has been described [64]. Furthermore, CD8 T cells expressing both 2B4 and CD160 were identified as a T-cell subset with a typical effector phenotype (i.e., CD27^−^CD45RA^+^CD56^+^CD57^+^) and expressing high levels of perforin and GrmB [62, 65].

Finally, TIM-3 is expressed at low levels on T_H_1 cells at a late stage of T-cell differentiation but not on T_H_2 cells, naïve T cells, B cells, macrophages, or dendritic cells. These evidences suggest that TIM-3 does not contribute to T-cell differentiation but has a role in the effector function of T_H_1 cells [66].

Also, during chronic viral infections, several inhibitory molecules are overexpressed on virus-specific T cells and this is associated with functional exhaustion. However, the expression of these molecules is also associated with the differentiation stage of T cells.

**Figure 2:**
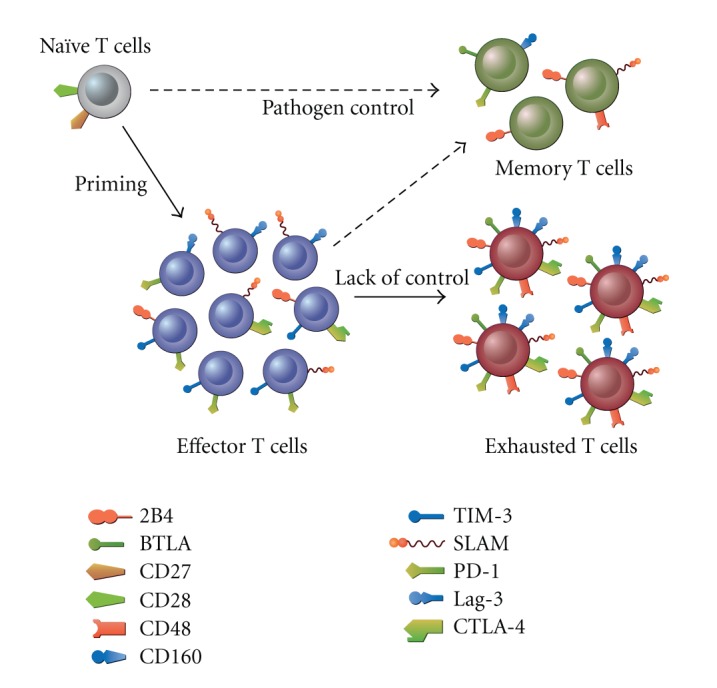
Expression of regulatory molecules following pathogen infection. Schematic overview of the pattern of expression of regulatory molecules. Following pathogen infection, key coinhibitory molecules are upregulated with different kinetics and play a role in regulating the development and the fate of effector T cells. In most cases, pathogens replication is controlled by the immune system leading to the contraction of effector T cells. Many different coinhibitory molecules, that is, PD-1, CTLA-4, BTLA, SLAM, and 2B4, play a role during this process. The remaining memory T cells (which are depending on the current models, derived either directly from naïve cells or from effector cells) express some coinhibitory molecules which depend on the type and biology of the pathogens. A hallmark of memory T cells is the lack of simultaneous expression of multiple coinhibitory molecules. Some regulatory molecules, however, are expressed by memory T cells, and this depends on the type of memory subset, that is, central or effector memory T cells. Conversely, when pathogens replication is not controlled, continuous stimulation of T cells, due to antigen persistence, prevents the full contraction of effector cells and leads to their functional exhaustion. A hallmark of exhausted effector cells is the simultaneous expression of several coinhibitory molecules. The simultaneous expression of these coinhibitory molecules is associated with their functional anergy.

We have recently (Viganò et al., personal observation) performed a comprehensive investigation of the expression of PD-1, 2B4, CD160, CTLA-4, TIM-3, Lag-3, and SLAM on CD4 and CD8 T-cell subsets identified according to their differentiation state (i.e., Naïve, memory, effector/memory). These analyses showed that whereas virtually none of the coinhibitory molecules tested was present on naïve cells, those were present on memory T cells but at low levels, and more importantly, that effector/memory T cells expressed a significantly higher density coinhibitory molecules simultaneously (Figure 2).

## 3. Role of Coinhibitory Molecules in the Contraction of the Effector Phase of Immune Responses

The immune system is able to mount strong and efficient immune responses against pathogens without damaging organs [67]. This is notably achieved by the induction of the contraction and termination of the immune response after control or elimination of the infectious agent. During the contraction, the majority of effector T cells die, while remaining cells survive as memory cells [24, 25]. The elimination of effector cells mainly occurs via apoptosis and a number of pro- and antiapoptotic molecules were shown to be involved in this process [68]. The contraction of the immune response and the determination of T-cell fate depend on many transcription factors regulated during the course of the immune response [69]. These factors can be either induced or repressed by different signaling pathways provided, with different strength and kinetics, by costimulatory and coinhibitory molecules [70, 71].

One of the best-established mechanisms involved in the regulation of TCR signaling is the interaction between costimulatory (CD28) and coinhibitory (CTLA-4) molecules with CD80 or CD86 expressed by dendritic cells (DCs). Cross-linking of CD28 on T cells synergizes with TCR signaling to induce activation. Conversely, cross-linking of CTLA-4 induces an inhibitory signal which prevents T-cell activation [26, 72]. CTLA-4 is upregulated on activated T cells and, as a structural homologous to CD28 with higher affinity for CD80 or CD86, it competes with CD28 to inhibit TCR signaling [26].

PD-1 is another well-known regulatory molecule. It is expressed on activated CD4 and CD8 T-cells, NKT, B cells, and activated monocytes [1, 73], and its expression is induced by TCR- and BCR-mediated signaling [74]. The two PD-1 ligands (i.e., PD-L1 and PD-L2) differ in their expression pattern [75]. PD-L1 (B7-H1, CD274) is expressed by a broad array of cells (e.g., vascular endothelial cells, epithelial cells, muscles cells, hepatocytes) whereas PD-L2 (B7-DC, CD273) expression is restricted to hematopoietic cell types (i.e., DC, macrophages, mast cells) [76]. PD-1/PD-Ls pathway regulates the balance between stimulatory and inhibitory signals needed for effective immune responses against pathogens [77–80]. Engagement of PD-1 by PD-L1 leads to the inhibition of CD28-mediated costimulation and thus of TCR-mediated lymphocyte proliferation and cytokines secretion. The relative levels of expression of inhibitory (PD-Ls) and stimulatory (CD80/CD86) ligands by antigen-presenting cells (APC) can determine the extent of T-cell activation while PD-L1 expression on nonlymphoid tissues may determine the extent of effector immune responses at sites of inflammation [77]. Also member of the B7-CD28 family, BTLA, is an inhibitory receptor able to recruit phosphatases to dampen TCR signaling [81] through the interaction with HVEM expressed on naïve T and B cells. HVEM-BTLA signaling was shown to limit T-cell activity *in vivo* and to negatively regulate homeostatic expansion of CD4 and CD8 T cells [82]. Finally, HVEM can also interact with CD160, resulting in an inhibitory signaling dampening T-cell activation [83].

## 4. Functional Exhaustion and Loss of Effector Functions

During chronic viral infections such as HIV and HCV, several inhibitory molecules are overexpressed on virus-specific CD4 and CD8 T cells and this is associated with a state of functional deficiency also called functional exhaustion. Exhaustion is characterized by the progressive loss of T-cell functionality, leading ultimately to the deletion of exhausted T cells. The loss of the distinct T-cell functions occurs sequentially [84]. IL-2 production and T-cell proliferation potential are lost first. TNF-*α* production and cytotoxic capacity disappear later followed, ultimately, by the loss of IFN-*γ* production. Finally, deeply exhausted T cells are deleted via apoptosis [84].

The current hypothesis is that functional exhaustion occurs as a consequence of the attempt of the immune system to limit the magnitude of effector T-cell responses in order to safeguard against autoimmune responses and inflammatory damages. Nonetheless, this mechanism of protection may compromise effective immunity against persistent infectious agents and tumors [85].

Functional exhaustion occurs in the context of persistent high antigenic load and was first described in mice during chronic lymphocytic choriomeningitis virus (LCMV) infection [86] where LCMV-specific CD8 T cells persisted during the chronic phase of infection but lacked cytotoxic potential. Nonfunctional (i.e., anergic) antigen-specific CD8 T cells were also observed in the context of SIV [87], HIV [88], hepatitis B virus (HBV) [89], HCV [90, 91], and human T-lymphotropic virus 1 (HTLV1) [92] virus infection as well as in patients with persistent tumors [93]. However, mechanisms leading to exhaustion including the fundamental differences between exhausted cells and terminally differentiated cells or senescent (replication incompetent) cells remain unclear [94].

PD-1 was the first inhibitory receptor associated with immune exhaustion [95] in the seminal study performed in the LCMV model [96]. RNA microarray analyses of exhausted LCMV-specific CD8 T cells showed a marked upregulation of PD-1 expression [96]. Multiple studies have confirmed that high expression levels of PD-1 are associated with functional anergy and increased susceptibility to apoptosis [88, 97] in the context of human virus infections such as HIV [88, 91, 97–99], HCV [90, 91], HBV [89, 100], and also established tumors [101–105]. Of interest, it was reported that blockade of PD-1 signaling *in vivo* and *in vitro* resulted in the restoration of HIV-specific CD8 T-cell proliferation capacity and IL-2 production [96, 106]. However, the functional restoration by PD1/PD-L blockade was incomplete, and defects in CD8 T cells remained [96], suggesting the involvement of additional negative regulatory pathways in T-cell exhaustion [107, 108]. Analyses of global gene expression profiles of exhausted CD8 T cells identified the involvement of many coinhibitory receptors [109]. More recently, the severity of LCMV infection was associated to the number and the intensity of coinhibitory receptors expressed by virus-specific CD8 T cells [107].

Among these molecules, TIM-3, Lag-3, 2B4, CTLA-4, CD160, BTLA, KLRG1, CD305, and CD200R have been further investigated in the context of several human chronic virus infections and established tumors. In particular, the coexpression of TIM-3 and PD-1 was observed on both CD4 and CD8 T cells from patients with HIV [110] or HCV [111–113] chronic infections and correlated with T-cell exhaustion and diseases progression. In addition, TIM-3- and PD-1-expressing CD8 T cells represented a major population within tumor-infiltrating lymphocytes (TILs) in several murine models of cancer and in the blood of patients with advanced melanoma [114, 115]. In all cases, TIM-3/PD-1-expressing cells represented the most impaired population of CD8 T cells. Of note, the blockade of both molecules could restore CD8 T-cell effector functions (proliferation potential and cytotoxic capacity) of antigen-specific CD8 T cells and was associated with the control of tumor growth [110–113, 115].

CTLA-4 is another coinhibitory receptor upregulated in the context of chronic infections [91] and tumors [116]. It has been shown that CTLA-4 was overexpressed on CD4, but not CD8, T cells of SIV-infected macaques [117] and HIV-infected patients [118, 119]. Furthermore, the combination of CTLA-4 blockade and 4-1BB (CD137) activation enhanced tumor rejection by increasing T-cell infiltration, proliferation capacity, and cytokines production [120].

Of interest, BTLA was reported to be persistently expressed by melanoma-specific CD8 T cells, thus inhibiting their antitumor function [121]. On the other hand, BTLA expression on CD4 and CD8 T cells decreased during HIV infection and this was associated with CD4 T-cell differentiation and activation [122, 123]. Enhancing BTLA pathway may therefore represent an alternative therapeutic strategy to overcome immune activation during chronic HIV infection.

Lag-3 is an activation-induced cell-surface molecule, whose overexpression during chronic virus infection is also commonly associated with T-cell exhaustion and functional impairment. Blocking of Lag-3 alone failed in rescuing T-cell function or in decreasing plasma viremia during chronic LCMV infection [108], while blockade of both PD-1 and Lag-3 synergistically improved T-cell responses and decreased viral loads *in vivo* [107]. Elevated levels of Lag-3 and CTLA-4 were found in PD1^+^ CD4 T cells from HIV-infected patients [124] and in tumor-derived NY-ESO-1-specific CD8 T cells [125]. Functionality of these T-cell subsets was more impaired than in Lag-3^−^PD-1^−^ or single Lag-3^+^ subsets [125].

SLAM family members are immunomodulatory receptors with a role in the regulation of costimulation, T-cell cytokines production, and cytotoxic activity. 2B4, which is a key molecule from this family, is involved in CD8 T-cell and NK-cell cytotoxicity. However, the proportion of 2B4^+^ CD8 T cells in HIV-infected patients correlated with immune activation of memory T cells and was increased in patients with progressive disease [126]. In addition, IFN-*γ* secretion and cytotoxic activity of 2B4^+^ CD8 T cells were significantly lower following stimulation with HIV as compared to influenza-derived antigens, respectively [127]. Furthermore, during infectious mononucleosis, the expression of SLAM and 2B4 on CD8 T cells correlated with severity of symptoms and viral loads [128].

The coexpression of molecules such as 2B4 and CD160, which have been related to potent cytolytic functions [62, 65], was associated with exhaustion and regulation of virus-specific CD8 and CD4 T cells in the context of chronic virus infections [83, 129]. A recent study showed a high frequency of CD8 T cells coexpressing PD-1, 2B4, CD160, KLRG1, LAG-3, and CTLA-4 in HCV infection. The coexpression of these molecules was associated with low levels of CD127 expression and correlated with impaired proliferation capacity [129].

The expression of another set of inhibitory molecules (i.e., PD-1, CTLA-4, CD305, and CD200R) has been investigated on CD4 T cells from HCV-infected patients. PD-1 and CTLA-4 were upregulated by HCV-specific CD4 T cells from patients with chronic infection, while CD305 and CD200R were upregulated in patients with cleared infection. Of note, the blockade of PD-Ls increased the expansion of CD4 T cells [130].

In the context of HIV infection, the presence of HIV-specific CD8 T cells coexpressing CD160, 2B4, and PD-1 but not Lag-3 was reported. The simultaneous expression of these molecules correlated with the level of virus replication and decreased cytokines production. The proliferative capacity was restored by blocking both PD-1/PD-L1 and 2B4/CD48 interactions [131]. Along the same line, another group showed that more than 30% of HIV-specific CD4 T cells expressed simultaneously PD-1, CTLA-4, and TIM-3, whereas less than 2% of CMV- or varicella-zoster virus-specific CD4 T cells coexpressed all three receptors. The coexpression of these molecules on HIV-specific CD4 T cells was more strongly correlated with the viral load compared with the expression of each receptor individually [132].

## 5. Potential Therapeutic Applications

The well-established immunosuppressive properties of coinhibitory molecules and the potential to revert exhausted or inactivated T-cell responses following selective blocking of their function made these markers interesting targets for therapeutic intervention in patients with persistent viral infections or cancer. To date, clinical and preclinical data are available for anti-CTLA-4 and anti-PD-1 blocking agents [87, 96, 133–136].

Initial human clinical trials assessing the effects of a blocking anti-CTLA-4 antibody demonstrated not only a reduction in tumor mass and clinical benefit in a minority of treated subjects but also an increase in systemic inflammation [137, 138]. Improvement in safety of these antibodies resulted in the recent approval by the U.S. Food and Drug Administration of a human monoclonal antibody against CTLA-4 (Ipilimumab, MDX-010, Yervoy) for the treatment of metastatic melanoma. In both early and late phase trials, Ipilimumab has demonstrated consistent activity against melanoma. However, serious (grade 3–5) immune-related adverse events occurred in 10–15% of patients. Thus, while providing a clear survival benefit, Ipilimumab administration requires careful patient monitoring combined to, sometimes, treatment with immune-suppressive therapy [133, 134]. In contrast, anti-CTLA-4 blockade failed to show benefit in terms of plasma viral load or survival in acutely or chronically SIV-infected macaques [139, 140]. Since CTLA-4 is preferentially upregulated on CD4 T cells and not on CD8 T cells [118], it might be possible that the blockade of anti-CTLA-4 induced an expansion and activation of CD4 T cells thus providing additional targets to HIV without significant improvement of CD8 T-cell functions.

Preclinical data showed how prevention of *in vivo* interactions between PD-1 and PD-L1 enhanced T-cell responses via the restoration of their ability to undergo proliferation, secrete cytokines, and lyse-infected cells and ultimately induce substantial reduction in viral loads. Of note, blockade of the PD-1/PD-L1 inhibitory pathway *in vivo* demonstrated a beneficial effect on CD8 T cells in mice that were lacking CD4 T-cell help [96]. This study identified a potentially effective immunotherapeutic strategy for chronic viral infections. This has then been further explored in nonhuman primates in a recent study evaluating the safety and immunomodulatory potential of an anti-PD-1 blocking antibody in SIV-infected macaques [87]. The treatment was well tolerated and led to a rapid increase in virus-specific CD8 T-cell responses with improved functional quality, both in peripheral and in GALT. PD-1 blockade also resulted in the expansion of virus-specific CD4 T cells, memory B cells, and higher titers of virus-specific antibodies. In contrast, one additional study showed an increase in CD4 T-cell activation and viral replication in mucosal sites [140]. Furthermore, a humanized anti-PD-1 monoclonal antibody (ONO-4538) is currently tested in a Phase 1 study in patients with recurrent or treatment-refractory cancer. Preliminary data support the safety, tolerability, and pharmacokinetic profile of a single-dose of the drug. In addition, preliminary evidences of antitumor activity were observed [135, 136].

There is currently a strong interest in the potential for clinical interventions targeting immunoregulatory networks to enhance immunity against cancer cells and persistent viruses or to boost the efficacy of preventive and therapeutic vaccines. The studies discussed previously have yielded promising results but have also highlighted important safety issues. This strongly indicates the importance to better understand mechanisms of immune regulation in order to exploit them for potential therapeutic applications.

## 6. Conclusion

Coinhibitory molecules are involved in maintaining the balance between the capacity to generate effector T cells able to control pathogens and the preservation of tolerance. During the development of immune responses, key coinhibitory molecules are upregulated with different kinetics and play a role in regulating the development and the fate of effector and memory T-cell responses. In most cases, pathogens replication is controlled by the immune system leading to the contraction of effector T cells. Many different coinhibitory molecules, that is, PD-1, CTLA-4, BTLA, SLAM, and 2B4, play a role during this phase. The remaining memory T cells express some coinhibitory molecules which depend on the type and biology of the pathogens and also on the level of differentiation. However, a hallmark of memory T cells is the lack of simultaneous expression of multiple coinhibitory molecules (Figure 2). Conversely, when pathogens replication is not controlled, continuous stimulation of T cells, due to antigen persistence, prevents the full contraction and leads to functional exhaustion of effector T cells. In contrast to memory T cells (see the aforementioned part), a hallmark of exhausted effector cells is the simultaneous expression of several coinhibitory molecules (Figure 2). The simultaneous expression of these coinhibitory molecules is associated with their functional anergy, also called exhaustion.

However, several evidences both *in vitro* and *in vivo* suggest that this anergic state can be reverted by blocking the interactions between coinhibitory molecules and their ligands. For this reason, coinhibitory molecules are now targets of preclinical and clinical studies aimed to identify new therapeutic strategies in the context of chronic infections and tumors. To date, only two coinhibitory molecules have been investigated in clinical trials, that is, PD-1 and CTLA-4, but recent evidences have underlined the importance of targeting multiple pathways in order to improve functional restoration. It is very likely that in the close future many additional targets will be assessed in preclinical and clinical studies.

In addition, while most studies focused their attention on the reversion of functional exhaustion, additional parallel strategies may be envisioned, such as the prevention of exhaustion in the context of therapeutic immunization.

Moreover, preventing/reverting exhaustion as a therapy for chronic conditions might be difficult to achieve notably for safety issues. On one hand, the prevention/reversion of exhaustion counteracts a physiological mechanism which is probably settled in order to avoid tissue damages and autoimmunity. On the other hand, restoration of functionality might not be sufficient since it will restore the functions of cells which failed to control the infection or to eliminate the pathogens. Therefore, it seems wise to plan to combine the functional restoration of T cells to other immunotherapeutic interventions.
